# Supplementary material for: Management of a Focal Introduction of ASF Virus in Wild Boar: The Belgian Experience
Source: Pathogens. 2023 Jan 17;12(2):152. doi: 10.3390/pathogens12020152 (PMC9961158; doi:10.3390/pathogens12020152)
Supplement: Supplementary file 1 [file pathogens-12-00152-s001.zip › pathogens-2120506-supplementary.pdf]

<u>Supplementary materials</u>

Adaptation of the EU and functional zoning from November 2018 to November 2020

The EU regulated zones (November 2018) have been adapted 4 times (January, February, March 2019 and January 2020) with an extension of Part II following the discovery of new positive cases outside the infected area. A fifth adjustment was performed in May 2020 with a reduction of Part II following the absence of positive cases for more than 15 months in the western part of Part II. Regulated Parts II and I were finally lifted in November 2020 leading to the recovery of the ASF free status for Belgium at an EU level. Each adaptation has been subject to legislative changes at both EU (Annex of the Commission Implementing Decision 2014/709/EU) and regional levels (Figure S1).
Supporting material Figure S1 – Adaptation of the EU (Part II in pink and Part I in blue) and management (Gaume and Ardenne, infected or white) zoning according to the detection of new ASF-positive cases. The publication dates of the EU and functional zoning are mentioned (UE Part I and Part II) and of the Decrees of the Walloon Government (RW AGW).

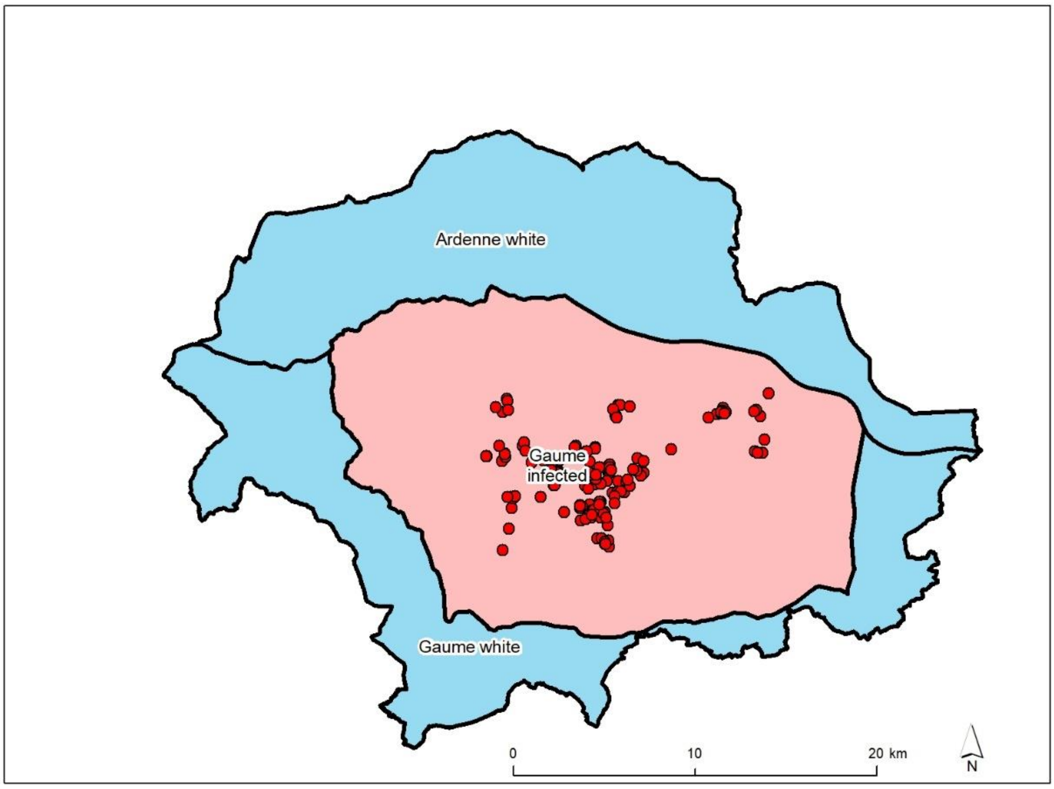

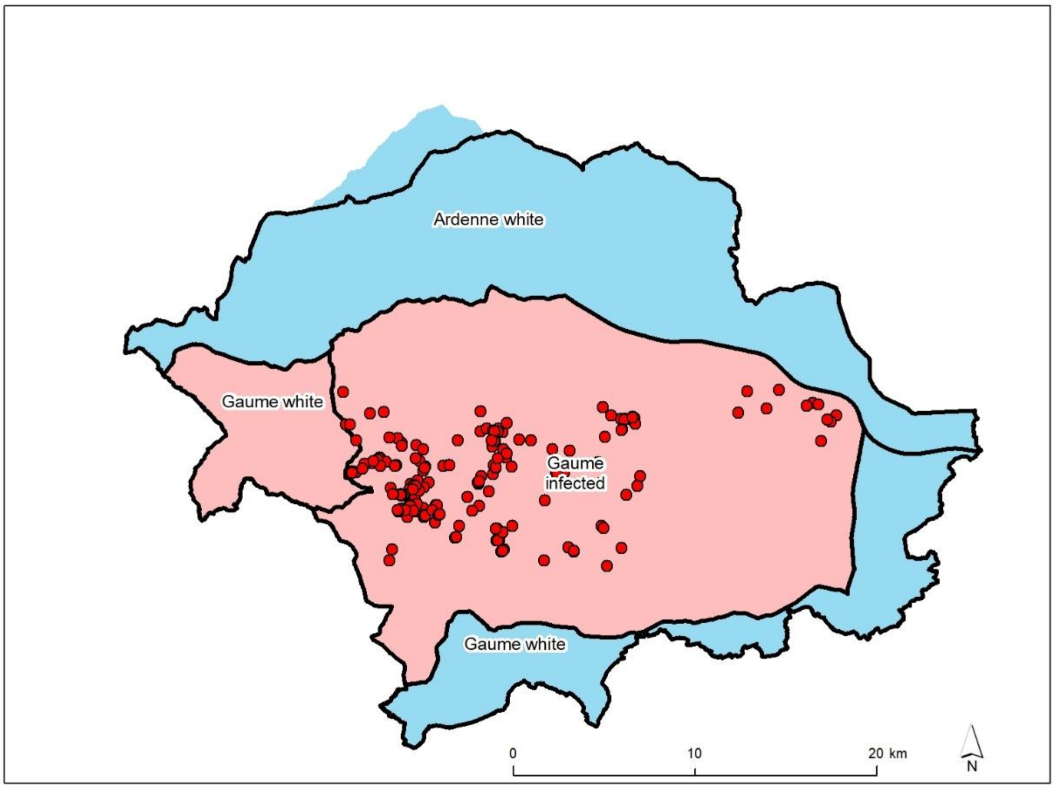
Ardenne white
Gaume white
Gaume infected
Gaume white
0
10
20 km
N

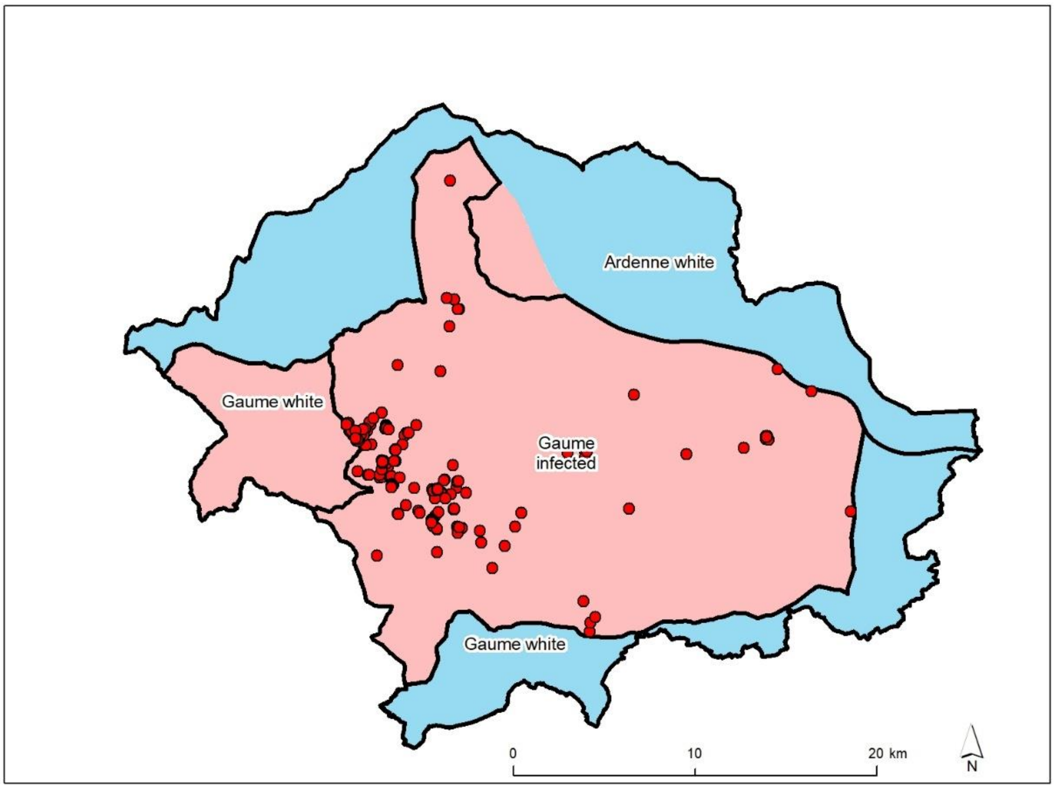
Ardenne white
Gaume white
Gaume infected
Gaume white
0
10
20 km
N

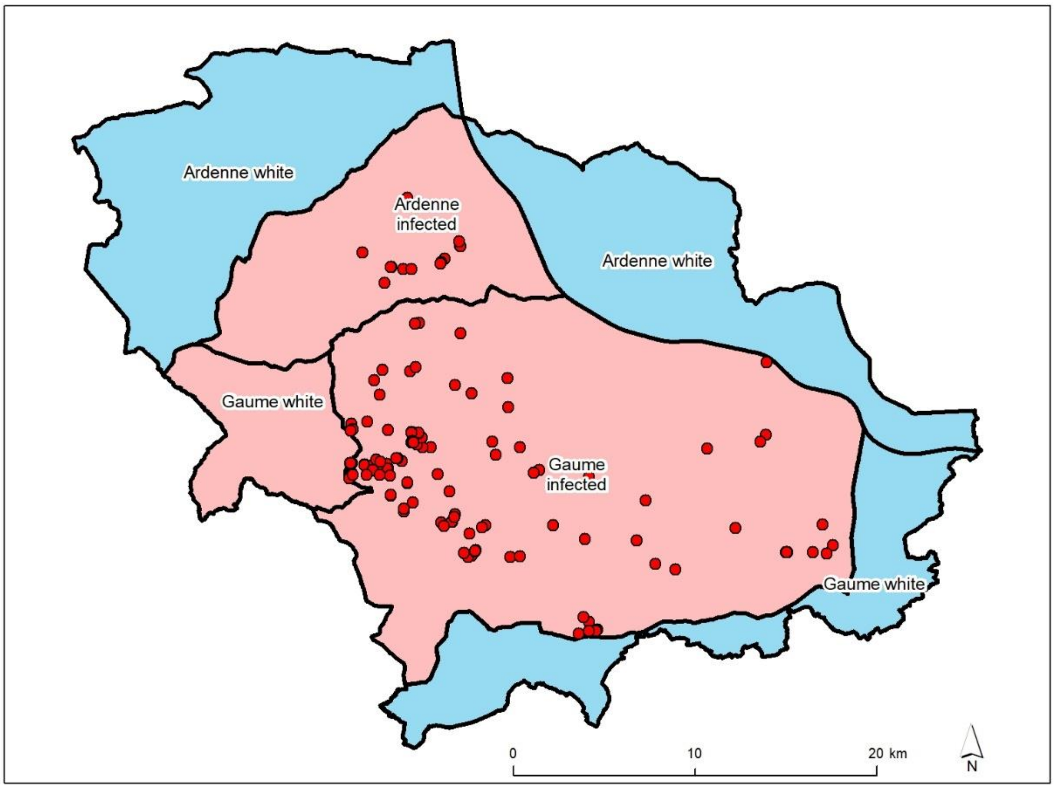
Ardenne white
Ardenne infected
Ardenne white
Gaume white
Gaume infected
Gaume white
0
10
20 km
N

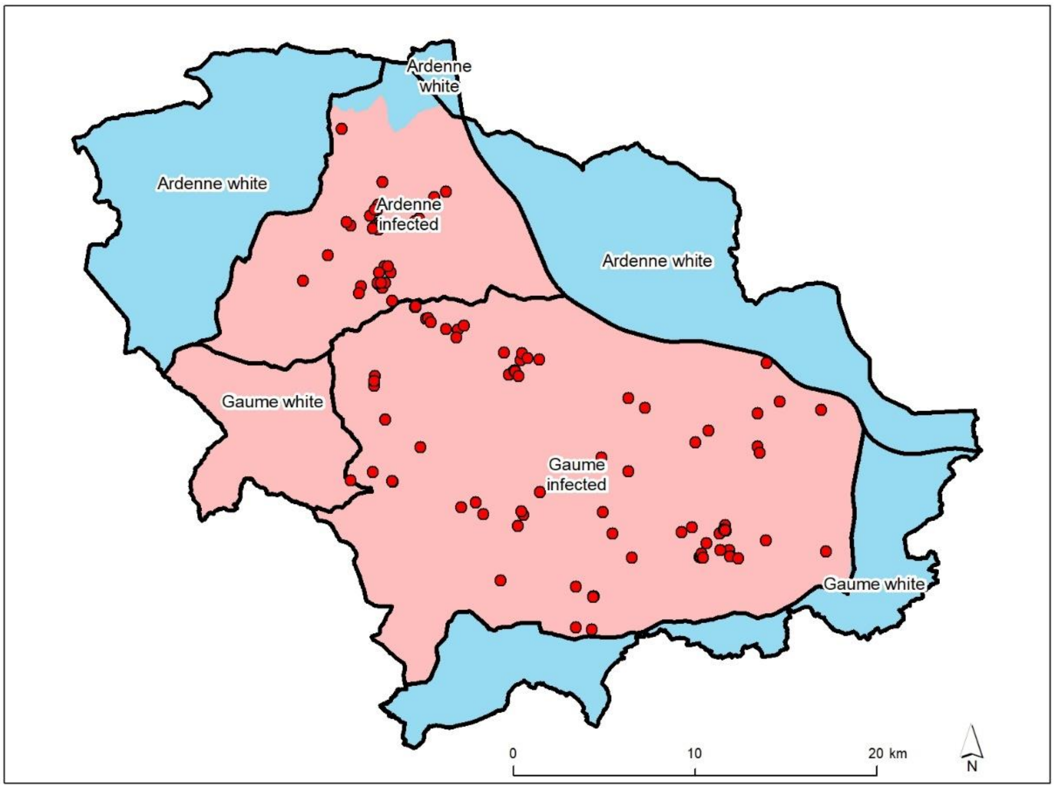
Ardenne white
Ardenne white
Ardenne infected
Ardenne white
Gaume white
Gaume infected
Gaume white
0
10
20 km
N

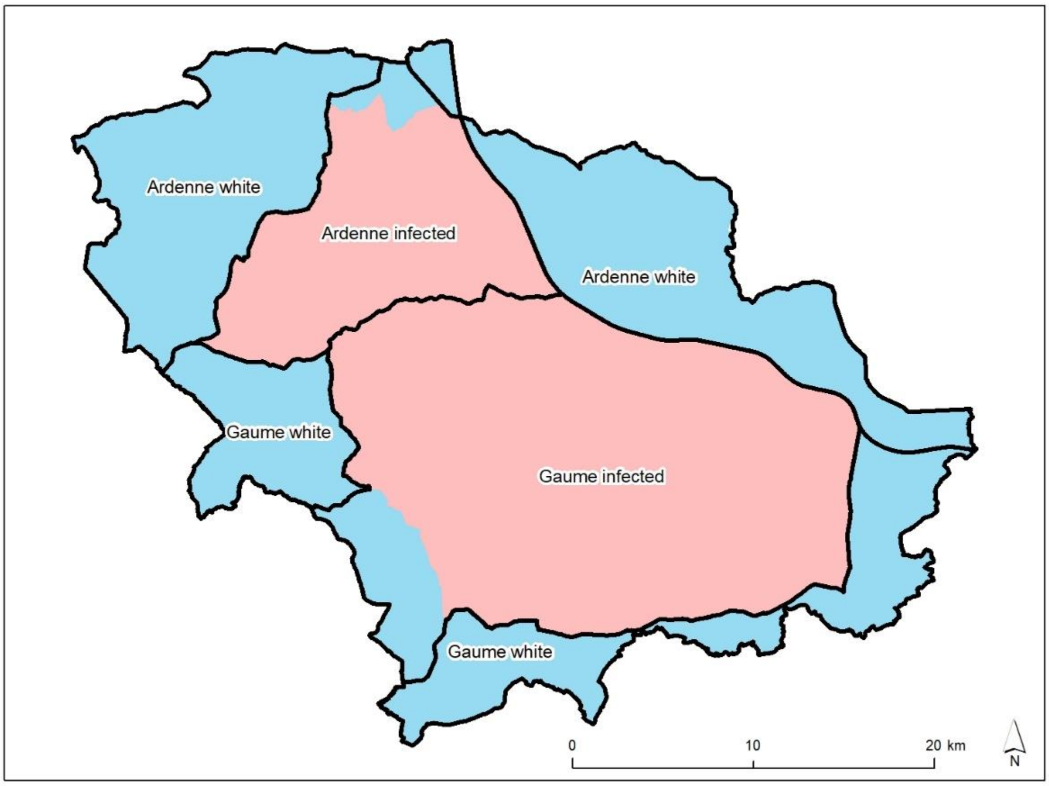

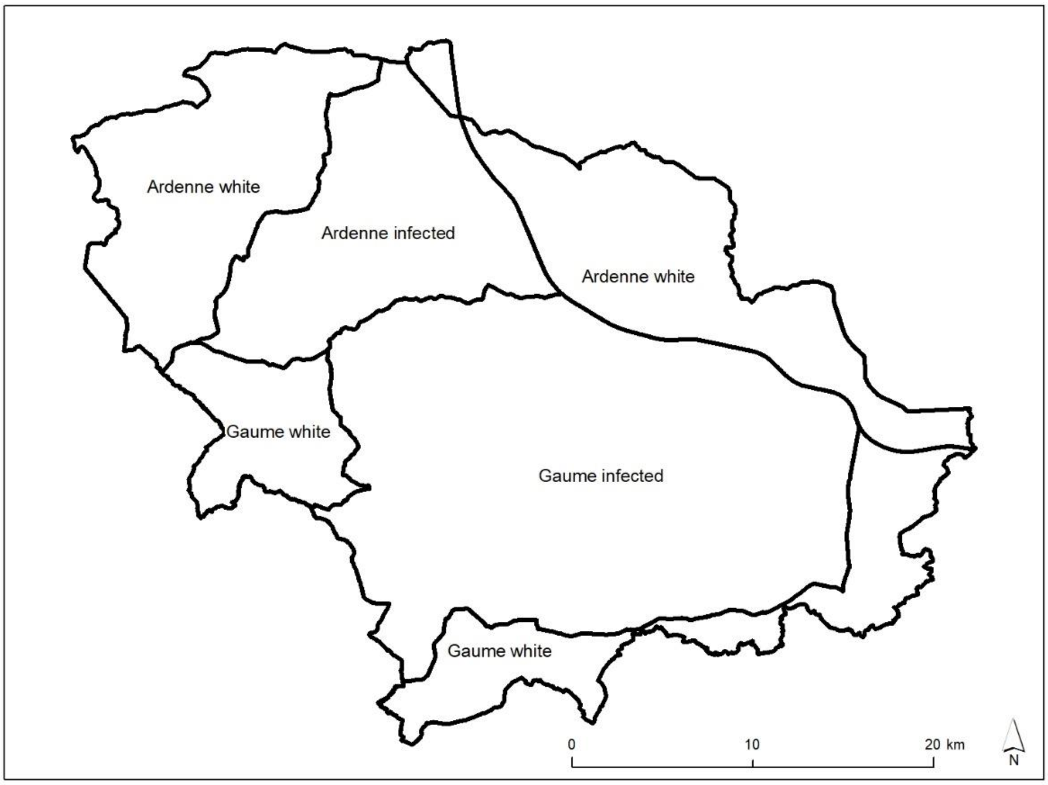

Figure S1. S1.1 UE Parts I & II 27/11/2018 - RW AGW 30/11/2018; S1.2 UE Parts I & II 22/01/2019 - RW AGW 11/01/2019; S1.3 UE Parts I & II 21/02/2019 - RW AGW 19/02/2019; S1.4 UE Parts I & II 25/03/2019 - RW AGW 19/03/2019; S1.5 UE Parts I & II 16/01/2020 - RW AGW 18/12/2019; S1.6 UE Parts I & II 15/05/2020 - RW AGW 18/12/2019; S1.7 RW AGW 18/12/2019.
